# HER2/neu-directed therapy for biliary tract cancer

**DOI:** 10.1186/s13045-015-0155-z

**Published:** 2015-05-29

**Authors:** Milind Javle, Chaitanya Churi, HyunSeon C. Kang, Rachna Shroff, Filip Janku, Rakesh Surapaneni, Mingxin Zuo, Christian Barrera, Humaid Alshamsi, Sunil Krishnan, Lopa Mishra, Robert A. Wolff, Ahmed O. Kaseb, Melanie B. Thomas, Abby B. Siegel

**Affiliations:** Department of Gastrointestinal (GI) Medical Oncology, Division of Cancer Medicine, The University of Texas MD Anderson Cancer Center, 1515 Holcombe Blvd. Unit Number 426, Room Number FC10.3062, Houston, TX 77030 USA; Department of Diagnostic Radiology, Division of Diagnostic Imaging, The University of Texas MD Anderson Cancer Center, Houston, TX USA; Department of Investigational Cancer Therapeutics, Division of Cancer Medicine, The University of Texas MD Anderson Cancer Center, Houston, TX USA; Scott & White Clinic, Temple, TX USA; Department of Radiation Oncology, Division of Radiation Oncology, The University of Texas MD Anderson Cancer Center, Houston, TX USA; Department of Gastroenterology, The University of Texas MD Anderson Cancer Center, Houston, TX USA; Gibbs Cancer Center & Research Institute, Spartanburg, SC USA; Departments of Medicine and Surgery, Columbia University, 622 West 168th Street, PH-14, room 105 C, New York, NY 10032 USA

**Keywords:** Receptor, ErbB-2, Gallbladder neoplasms, Cancer of the biliary tract

## Abstract

**Background:**

Biliary cancers are highly aggressive tumors that are often diagnosed an advanced disease stage and have a poor outcome with systemic therapy. Recent efforts towards molecular characterization have identified a subset of biliary patients that have *HER2/neu* amplification or mutation. *HER2/neu* amplification is associated with response to HER2/neu-directed therapy in breast and gastric cancers. However, the efficacy of HER2/neu-targeted therapy in biliary cancers is unknown.

**Patients and methods:**

We retrospectively reviewed cases of advanced gallbladder cancer and cholangiocarcinoma with *HER2/neu* genetic aberrations or protein overexpression who received HER2/neu-directed therapy between 2007 and 2014. Clinical data were retrieved from medical records, and imaging studies were independently reviewed.

**Results:**

Nine patients with gallbladder cancer and five patients with cholangiocarcinoma had received HER2/neu-directed therapy (trastuzumab, lapatinib, or pertuzumab) during the study period. In the gallbladder cancer group, *HER2/neu* gene amplification or overexpression was detected in eight cases. These patients experienced disease stability (*n* = 3), partial response (*n* = 4), or complete response (*n* = 1) with HER2/neu-directed therapy. One patient had *HER2/neu* mutation and experienced a mixed response after lapatinib therapy. The duration of response varied from 8+ to 168 weeks (median 40 weeks), and three patients are still on therapy. One patient developed *HER2/neu* amplification as a secondary event after FGFR-directed therapy for *FGF3-TACC3* gene fusion. The cholangiocarcinoma cases treated in this series had a higher proportion of *HER2/neu* mutations, and no radiological responses were seen in these patients despite HER2/neu-directed therapy.

**Conclusions:**

HER2/neu blockade is a promising treatment strategy for gallbladder cancer patients with gene amplification and deserves further exploration in a multi-center study.

## Introduction

Gallbladder cancer is one of the most aggressive solid tumors and represents an important cause of cancer-related mortality in South America and South Asia, while occurring less frequently in the United States and Western Europe. Late stage at presentation, early occurrence of liver and peritoneal metastases, and poor outcome with standard chemotherapy contribute to the poor prognosis of gallbladder cancer. Understanding the molecular characterization of this disease may help alter the dismal outcome with targeted therapeutics [1, 2]. An estimated 12–15 % of gallbladder cancers have *HER2/neu* amplification or protein overexpression using commonly accepted criteria for positivity [2–4]. Cholangiocarcinoma is increasing in incidence in the Western world. Recent efforts at molecular characterization have identified distinct subsets with prognostic and therapeutic implications [5]. *HER2/neu* mutation or amplification has also been reported in cholangiocarcinoma [6–8]. These patients may be candidates for HER2/neu-directed therapy.

The *HER2/neu* gene is located on the 17q12-q21 chromosomal region and acts as an oncogene in several human cancers [9]. HER2/neu protein overexpression, either as the product of gene amplification or transcriptional deregulation, is observed in approximately 20 % of breast and ovarian cancers and 12 % of gastric cancers [10]. The overexpression and amplification of *HER2/neu* has also been demonstrated in gastricesophageal and endometrial cancer, and in these tumors, it is also usually associated with a worse prognosis [11–14]. The HER2/neu receptors after dimerization can transactivate a number of downstream pathways like RAS-RAF-MEK-ERK1/2 or PI3k-AKT-mTOR resulting in cancer cell proliferation and cell survival [15, 16].

Several therapeutic agents have been developed for HER2/neu positive breast cancer: these include monoclonal antibodies such as trastuzumab and pertuzumab, small molecule tyrosine kinase inhibitors like lapatinib, and chemo-immunotherapy conjugates like ado-trastuzumab emtansine (T-DM1). These have substantially changed the management of breast and gastric cancers and have already been incorporated into standardized treatment algorithms [11, 17, 18]. However, data in regards to the efficacy of HER2/neu targeted therapy in biliary cancers are scarce.

## Patients and methods

### Case selection

All patients selected had pathologically confirmed gallbladder or biliary adenocarcinoma, had received HER2/neu-directed therapy, and had a minimum of 3 months of follow-up. Clinical, pharmacy, and pathology records were reviewed to identify cases that were HER2/neu positive and had received HER2-directed therapy. The study was approved by the Institutional Review Board of MD Anderson Cancer Center. Patient demographics, clinical data, survival data, and treatment history were retrieved from medical records.

### Tumor samples

Paraffin embedded blocks were sectioned, and hematoxylin and eosin (H&E) stained slides were reviewed by surgical pathology to confirm the tumor content in each section. Ten serial sections (4 μm) were cut from selected tissue blocks and areas with tumor tissue were macrodissected from those slides using the H&E slides as templates.

### DNA extraction

The pathologic diagnosis of each case was confirmed on routine H&E slides. All samples sent for DNA extraction contained a minimum of 20 % DNA derived from tumor cells. DNA was extracted from 40 mm of fresh frozen, paraffin-embedded (FFPE) tissue using the Maxwell 16 FFPE Plus LEV DNA Purification kit (Promega Corporation, Madison, WI, USA) and quantified using a standardized PicoGreen fluorescence assay (Invitrogen, Carlsbad, CA, USA).

### Next-generation sequencing (NGS)

For subjects with adequate biopsy tissue, an amplicon library was generated from 10 ng of DNA from each sample using the Ion Ampliseq Cancer Panel (Life Technologies, Carlsbad, CA, USA). FFPE cell pellets of the H2122 cell line diluted in the HL60 cell line were used as control. The 46 genes in the panel for detection of targetable mutations included the following: *AKT1, BRAF, FGFR1, GNAS, IDH1, FGFR2, KRAS, NRAS, PIK3CA, MET, RET, EGFR, JAK2, MPL, PDGFRA, PTEN, TP53, FGFR3, FLT3, KIT, ERBB2, ABL1, HNF1A, HRAS, ATM, RB1, CDH1, SMAD4, STK11, ALK, SRC, SMARCB1, VHL, MLH1, CTNNB1, KDR, FBXW7, APC, CSF1R, NPM1, SMO, ERBB4, CDKN2A, NOTCH1, JAK3,* and *PTPN11*. Primers for PCR amplification included the 190-primer pair pool provided by the vendor with an additional primer pair that was custom added to cover the “hotspot” location on codon 17 of AKT1. Following PCR amplification of target sequences, barcodes were ligated to the amplicons using the Ion Xpress Barcode Adaptors Kit (Life Technologies, Carlsbad, CA, USA). Library quantification was then performed using the Bioanalyzer High Sensitivity DNA Chip (Agilent Technologies, Santa Clara, CA, USA). The library was diluted in nuclease-free water to obtain a final concentration of 16 pM. Emulsion PCR was performed manually using the Ion Xpress Template Kit (Life Technologies, Carlsbad, CA, USA) followed by manual breaking of the emulsion to isolate the ion spheres (ISPs). The quality of the DNA following PCR was measured using the Qubit IonSphere Quality control kit (Life Technologies, Carlsbad, CA, USA).

Selective ISPs with DNA were isolated and sequenced on an Ion 316 Chip (four samples/chip) or a Ion 318 Chip (eight samples/chip) using the vendor-provided sequencing kit (Life Technologies, Carlsbad, CA, USA). Successful sequencing of a sample required at least 300,000 reads with a quality score of AQ20 (1 misaligned base per 100 bases). For a wild-type call, a minimum coverage of 250× was required. As tumor specimens were admixed with normal tissue, a minimum coverage of 500× with at least 10 % frequency was used as cutoff for a variant to be considered true. All variants detected by Ion PGM with at least 10 % frequency were selected for confirmation by alternate platforms. Further details regarding the methodology and analysis have been described previously [19]. For four samples, NGS was performed by Foundation Medicine using the Illumina Hiseq 2000 Platform (Illumina, San Diego, CA, USA) for 236 targetable GAs; the methods have been described previously [20, 21].

### Immunohistochemistry for HER2/Neu

Tissues were fixed in formalin and embedded in paraffin using standard techniques. The 4-μm-thick histologic sections obtained from the TMAs were deparaffinized and hydrated in decreasing alcohol concentrations. Antigens were recovered by exposure to microwaves in citrate buffer (pH 6.0) and washed in PBS (pH 7.4). The monoclonal antibody anti-ErbB2 (NCL-CB11; Novocastra, Leica Biosystems, Wetzlar, Germany) was used at a dilution of 1:40. The primary antibody was incubated at room temperature for 60 min and then incubated with the complex Super Picture Polymer Detection Kit (Zymed Laboratories Inc., San Francisco, CA, USA) in a Dako autostainer (Dako, Glostrup, Denmark). Standard criteria for HER2/Neu scoring were used [3]. Fluorescent in situ hybridization (FISH) was performed using methods. Scoring was conducted according to the College of American Pathologists/American Society of Clinical Oncology (CAP/ASCO) criteria for breast cancer [22].

## Results

**Fig. 1:**
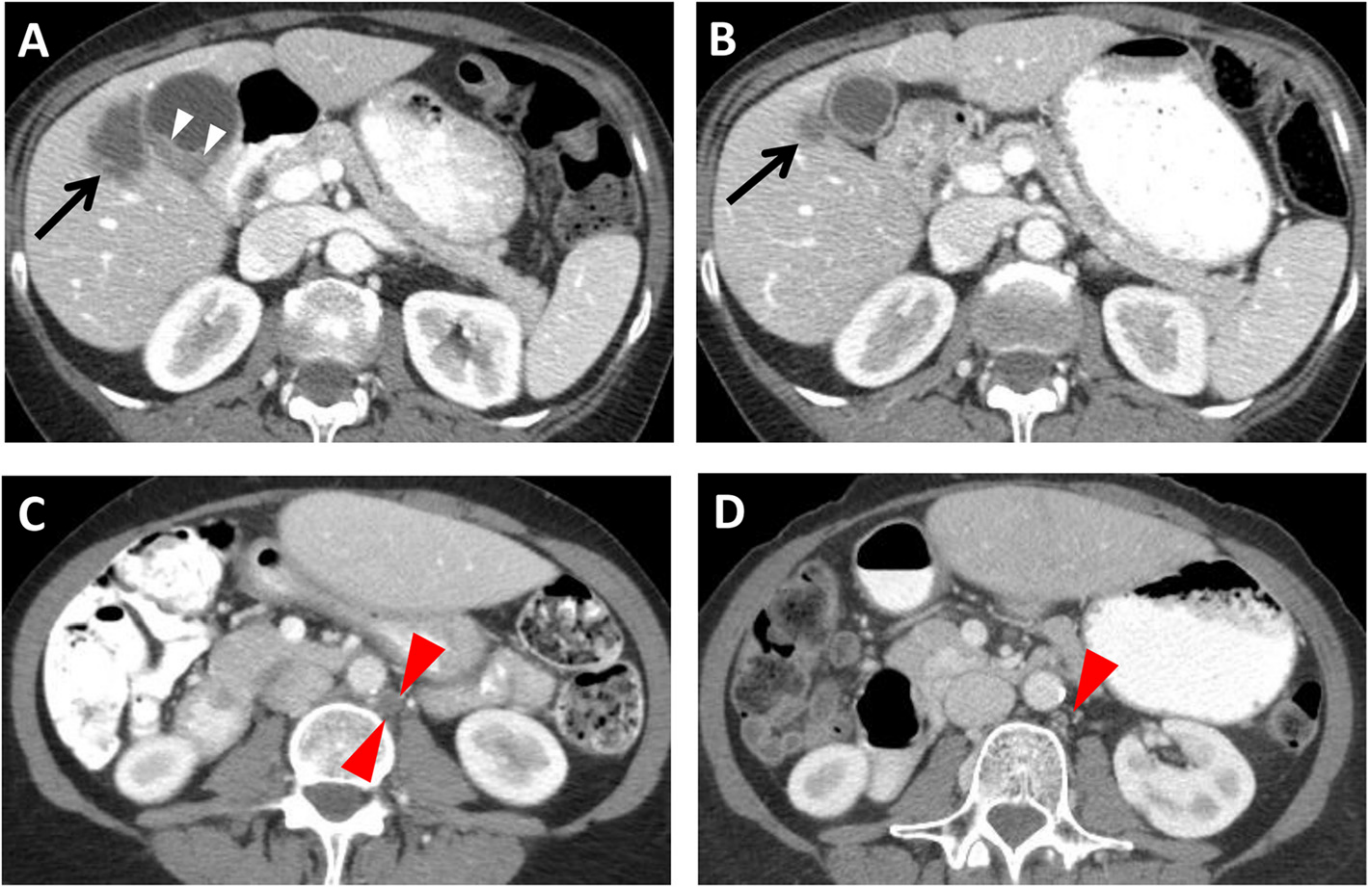
A 61-year-old female with gallbladder carcinoma invading the liver. Axial contrast-enhanced CT images demonstrate **a** a 2.4 × 1.3 cm polypoid mass (*small arrowheads*) in the gallbladder neck causing gallbladder obstruction. The mass directly invades the liver, with a 2.9 × 3.9 cm liver mass (*arrow*). **b** After 3 months of trastuzumab and FOLFOX, the polypoid gallbladder mass is no longer visualized, and the liver mass decreased to 1.2 × 1.1 cm (arrow). The patient was then treated with *en bloc* cholecystectomy and extended right hepatectomy, followed by capecitabine and trastuzumab for 7 months. **c** After 6 months of observation (14 months after surgery), the patient had small volume recurrence to retroperitoneal lymph nodes (*large arrowheads*). After treatment with FOXFOX and trastuzumab for 3 months, **d** a previously seen 1-cm retroperitoneal lymph node is nearly imperceptible

**Fig. 2:**
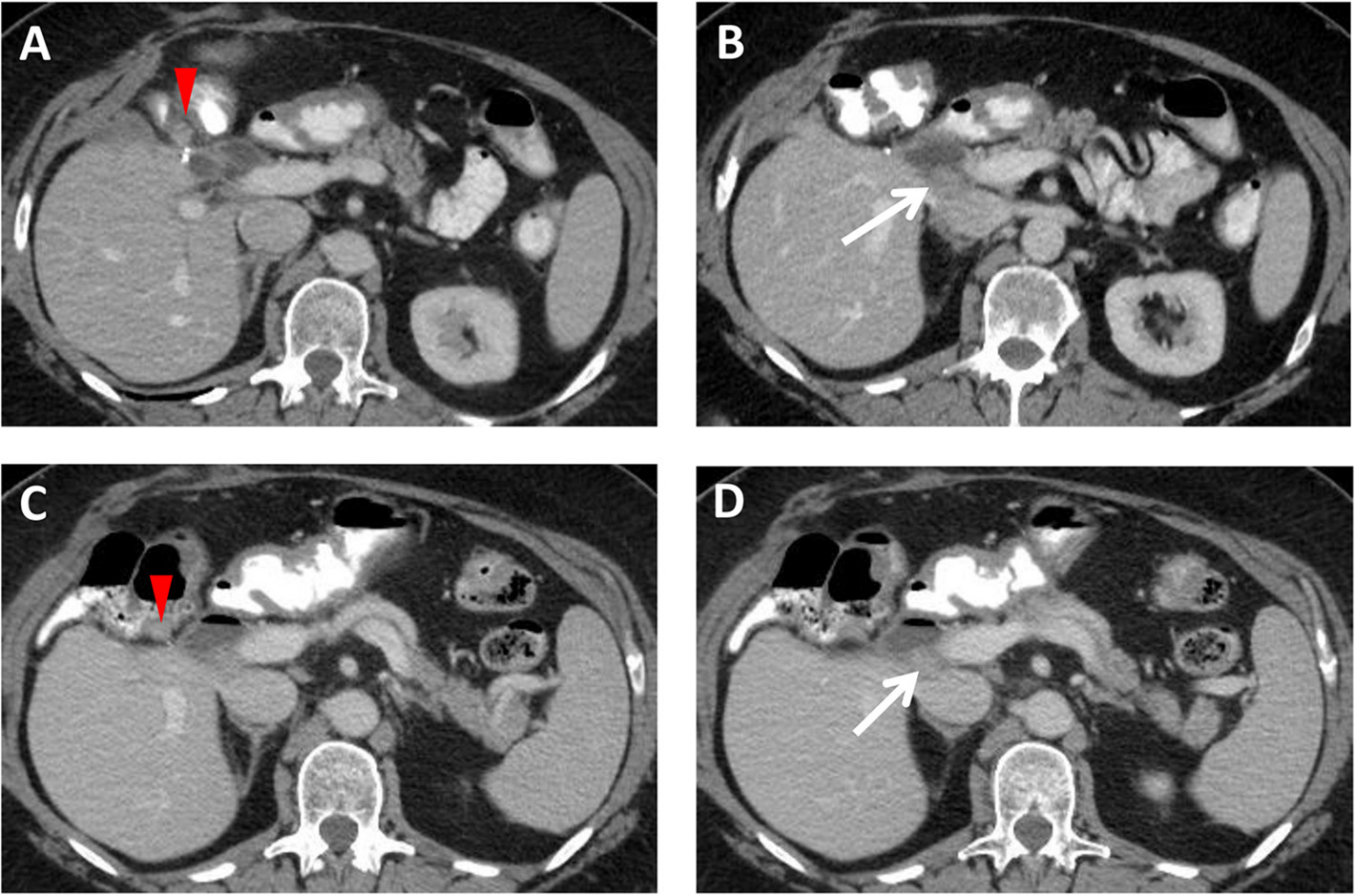
A 64-year-old female with recurrent gallbladder carcinoma. Axial contrast-enhanced CT images demonstrate **a** a 1.2-cm nodule (*arrowhead*) in the gallbladder fossa adjacent to the hepatic flexure and **b** a 1.7-cm nodule (*arrow*) in the portocaval region. Both nodules were new from the postoperative scan (following resection of recurrent tumor in the gallbladder fossa), in keeping with recurrence. **c**, **d** Thirteen months later, both nodules are stable after treatment with trastuzumab

**Fig. 3:**
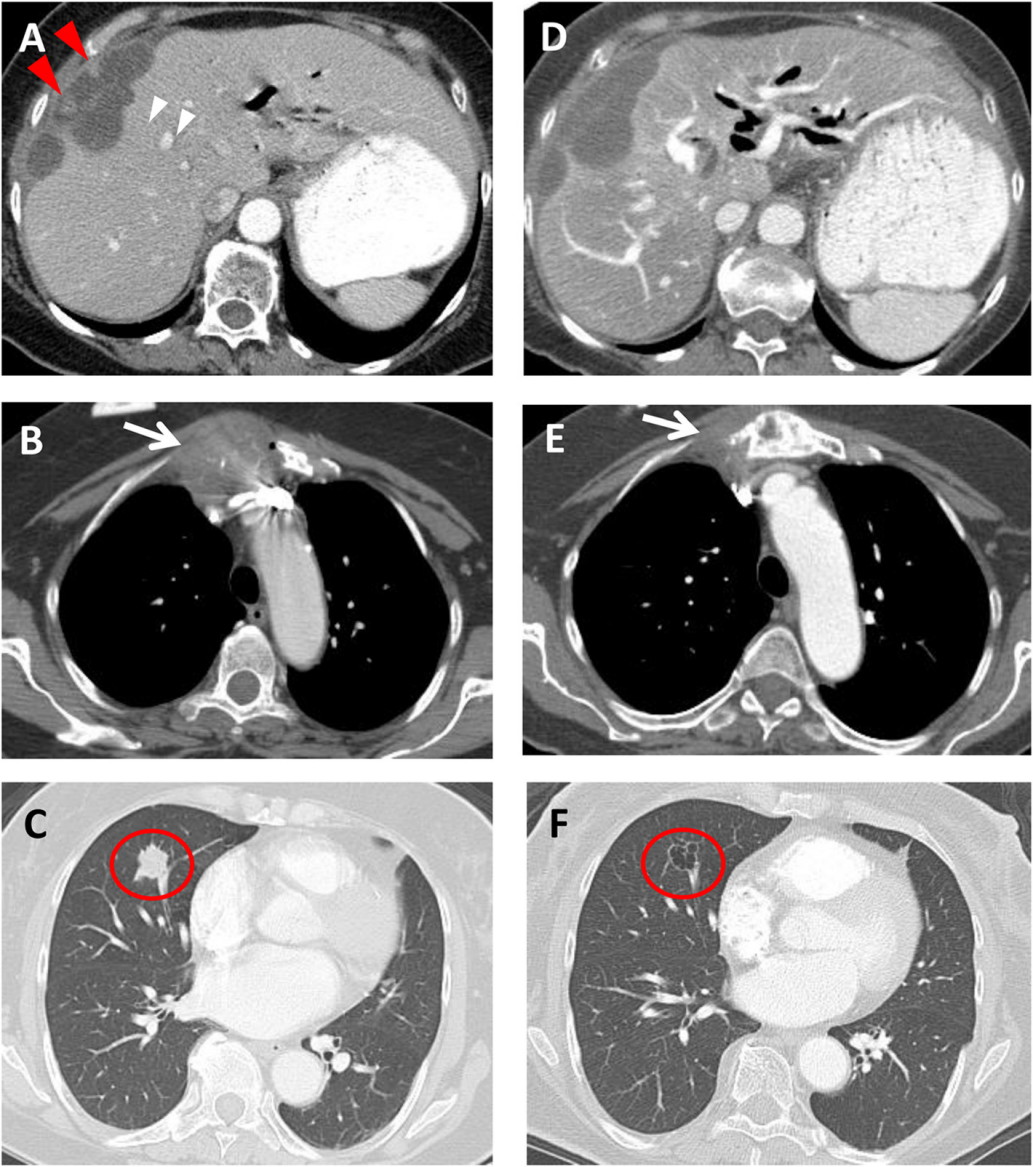
A 62-year-old female with recurrent metastatic gallbladder carcinoma with carcinomatosis. Axial contrast-enhanced CT images demonstrate **a** enhancing perihepatic nodules (*arrowheads*), **b** a 4.8-cm metastasis destroying the sternum (arrow), and **c** a 1.8-cm lung metastasis (*circle*). After 3 months of trastuzumab, **d** the perihepatic nodules are no longer visualized. **e** The sternum has become sclerotic with decreased size of the metastasis (*arrow*), and **f** the lung metastasis has undergone cavitation (*circle*).

**Fig. 4:**
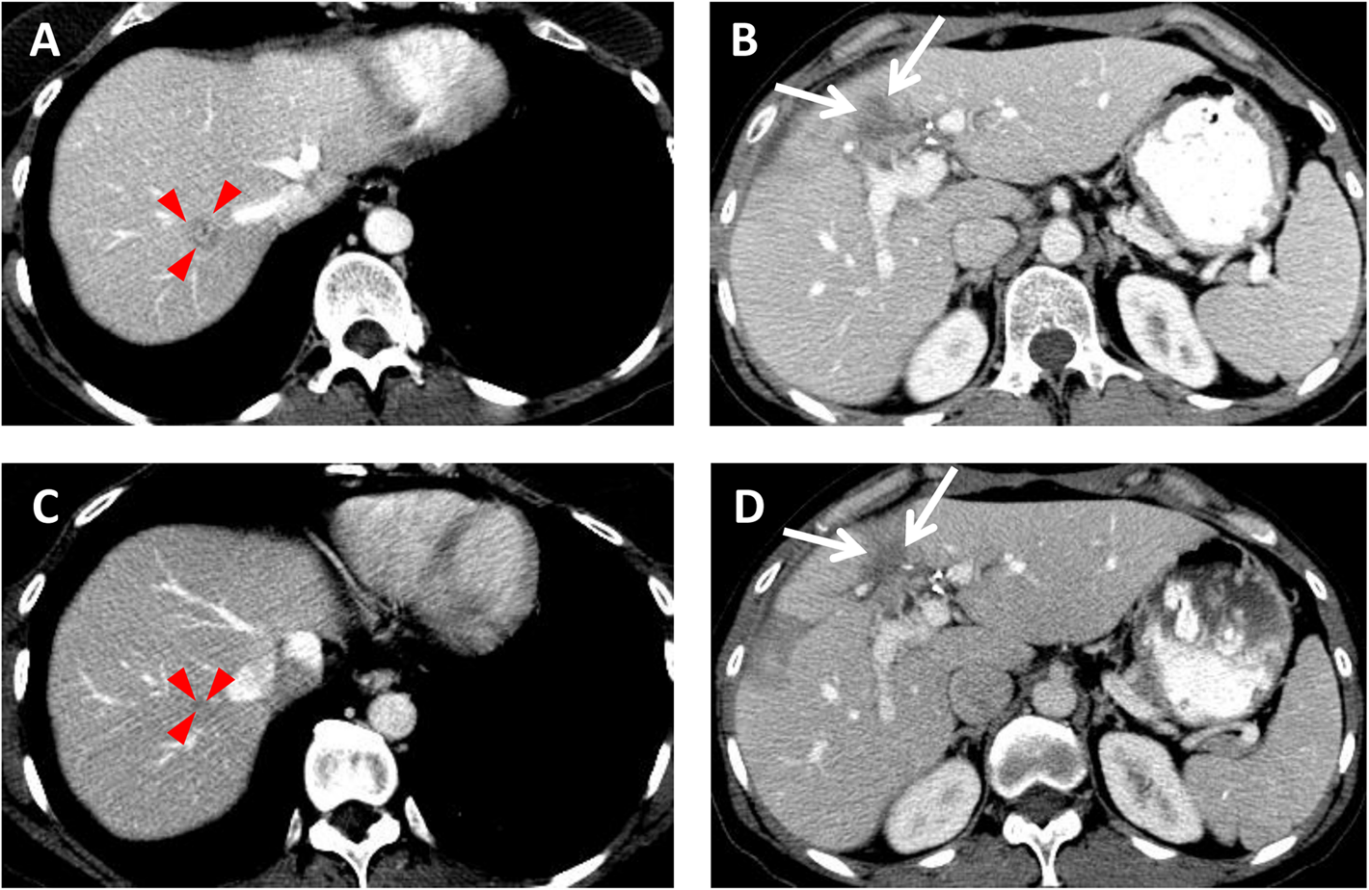
A 45-year-old female with metastatic gallbladder carcinoma. Axial contrast-enhanced CT images demonstrate **a** a 1.5-cm liver metastasis (*arrowheads*) and **b** prominent soft tissue (*arrows*) in the cholecystectomy bed abutting the liver. After 2 months of treatment with trastuzumab, **c** the liver metastasis is barely visible at 4 mm, and **d** the soft tissue mass in the resection bed, representing recurrent tumor, is decreased. She was stable for an additional 5 months, then had recurrence in the resection bed

In the gallbladder cancer group, there were seven females and two males, with a median age of 63 years. One patient was Asian while others were Caucasian (Table 1). All had metastatic disease; seven received trastuzumab either alone (*n* = 2) or in combination with chemotherapy (*n* = 5). Other HER2-directed therapies were lapatinib and pertuzumab. One patient who received lapatinib had a mixed response; this patient had a HER2/neu mutation V777L. All other cases had *HER2/neu* amplification or overexpression. In all these cases, trastuzumab was associated with partial response (*n* = 4), stable disease (*n* = 3), or complete response (*n* = 1). (Figures 1, 2, 3, and 4 illustrate the responses seen in this group). The duration of response varied from 8+ to 168 weeks (median 40 weeks), and three patients are still on therapy. In two cases, therapy was administered neoadjuvantly for T4 disease and was followed by surgical resection. In one case, disease recurrence occurred 14 months after surgical resection, which was again successfully salvaged with trastuzumab (Fig. 1). Details regarding prior therapy administered, concurrent chemotherapy, and duration of treatment are described in Table 2.

**Table 1:** Patient characteristics

Characteristics	GBCA (*n* = 9)	CCA (*n* = 5)
Sex		
Male	2	5
Female	7	0
Age (years)		
20–39	0	2
40–59	3	1
≥60	6	2
Ethnicity		
Asian	1	0
White	8	5
Tumor differentiation		
Poor	1	1
Moderate	8	4

**Table 2:** Gallbladder cancer: prior therapy , concurrent chemotherapy, and treatment duration

Pt #	Sites of disease	Prior therapy	HER2/neu test	HER2/neu therapy	Concurrent therapy	Duration of therapy (weeks)	Overall survival (weeks)	Best response
1	Bone, liver	Nil	IHC 3+	Trastuzumab	Gemcitabine, cisplatin	7	20	SD
2	Peritoneum, lung, liver	Gemcitabine + cisplatin, FOLFIRI + erbitux	Mutation (NGS) V777L	Lapatinib	Sirolimus	15	19	MR
3	Retroperitoneal LN, liver	Nil	FISH amplification	Trastuzumab	Nil	38	113	CR
4	Liver	Gemcitabine + cisplatin, capecitabine, FOLFOX	ERBB2 NGS amplification	Trastuzumab	Gemcitabine + irinotecan	40	62	PR
5	Liver, LN	Gemcitabine + cisplatin	ERBB2 NGS amplification	Trastuzumab	FOLFOX capecitabine	92	92	PR
6	Sternum, pleura, lung	Gemcitabine, capecitabine	AMPLIFIED (FISH)	Trastuzumab		168	178	PR
7	Retroperitoneal LN, celiac LN	Nil	AMPLIFIED (FISH)	Trastuzumab	Gemcitabine + cisplatin	22+	22+	PR
8	Retroperitoneal LN, supraclavicular LN	Gemcitabine + capecitabine, gemcitabine + cisplatin, pazopanib, dovitinib	ERBB2 NGS amplification	Trastuzumab + pertuzumab	Nil	8+	8+	SD
9	Lungs, brain	Gemcitabine + cisplatin radiation	IHC 3+	Trastuzumab	Paclitaxel capecitabine	72	96+	PR

HER2-positive tumors IHC 3+, FISH *HER2*/centromere 17 ratio ≥2.0 or both

*SD* stable disease, *MR* mixed response, *PR* partial response

**Fig. 5:**
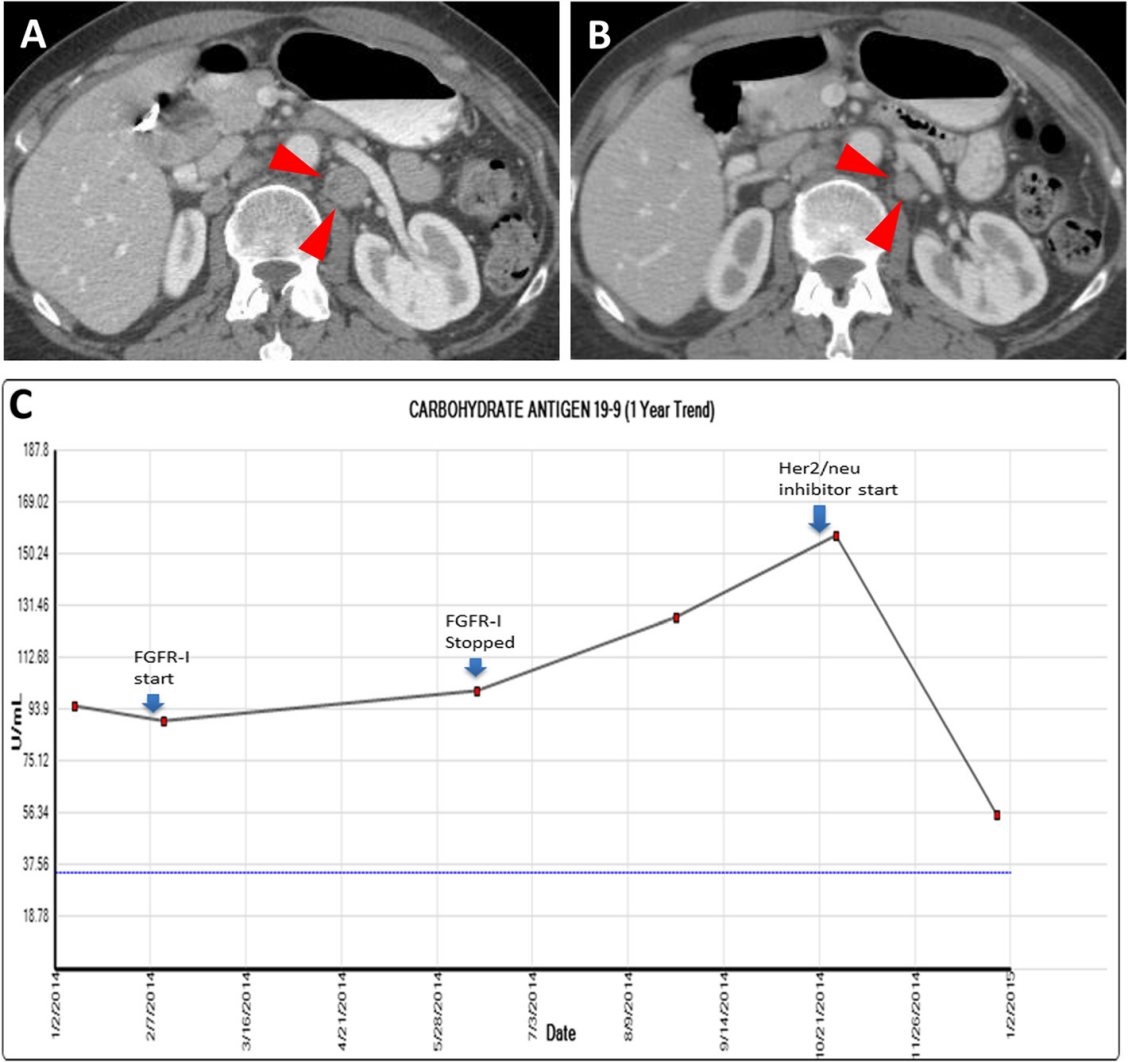
A 73-year-old female with metastatic retroperitoneal lymphadenopathy from gallbladder carcinoma. Axial contrast-enhanced CT images demonstrate **a** a 1.9-cm lymph node (arrowheads) posterior to the left renal vein. After 2 months of trastuzumab and pertuzumab, **b** the lymph node decreased to 1.2 cm. **c** Her CA 19-9 response to HER2/neu inhibition after prior FGFR inhibitor therapy

One patient had FGF3-TACC3 fusion gene and had received prior therapy with the FGFR-directed agents pazopanib and dovitinib. Repeat biopsy after these therapies indicated that the tumor had developed HER2/neu amplification as a secondary event, while this was absent at initial diagnosis. This finding was confirmed by FISH analysis of the tumor biopsy before and after the FGFR-directed therapy. In this case, disease stability or response was noted after FGFR-directed therapy as well as from trastuzumab. This was also accompanied by a CA19-9 response (Fig. 5a–c).

In the cholangiocarcinoma group, the median age was 59 years; all patients were Caucasian with stage IV disease. Two patients had *HER2/neu* mutations (V777L and S310F) while the others had amplification using FISH or NGS (Table 3). Associated mutations noted on NGS along with response data are depicted in Table 4. All but one had received prior therapy, and two patients received no concurrent chemotherapy. In case of cholangiocarcinoma, no responses occurred from trastuzumab therapy, unlike the case with gallbladder cancer. In all cases, HER2/neu-directed therapies were well-tolerated without any notable cardiac events despite the length of therapy.

**Table 3:** Cholangiocarcinoma: prior therapy , concurrent chemotherapy, and treatment duration

Pt #	Sites of disease	Prior therapy	HER2/neu test	HER2/neu therapy	Concurrent therapy	Duration of therapy	Overall survival	Best response
1	Liver, mediastinum, Lung	Gemcitabine folfirinox	Mutation (NGS) V777L	Trastuzumab	Gemcitabine, docetaxel	19	29	PD
2	Liver, peritoneum, lung	Gemcitabine + cisplatin	ERBB2 amp NGS	Trastuzumab	FOLFOX	14	25	PD
3	Mediastinum	Gemcitabine + cisplatin, FOLFIRI, GTX	ERBB2 NGS S310F	Trastuzumab	FOLFOX	7	7	PD
4	Liver, lung	Nil	AMPLIFIED (FISH)	Trastuzumab		6	8	PD
5	Lung, liver, bones	Gemcitabine + cisplatin	AMPLIFIED (FISH)	Trastuzumab		10	12	PD

HER2-positive tumors (IHC 3+, FISH *HER2*/centromere 17 ratio ≥2.0 or both

*SD* stable disease, *PD* progressive disease, *CR* complete response, *PR* partial response

**Table 4:** Associated mutations noted on NGS along with response data

Cancer type	HER2/neu status	Associated mutations	Best response	Agent	PFS (weeks)	OS (weeks)
Cholangioca	Mutation (V777L)	FGFR3, TP53	SD	Trastuzumab	19	29
Cholangioca	Amplification	BAP1, CDKN2A, KDM6A, PBRM1, SETD2	PD	Trastuzumab	14	25
Cholangioca	Mutation (S310F)	KRAS, MYC, TP53, EZH2, MSH6	PD	Trastuzumab	7	7
Cholangioca	Amplification		PD	Trastuzumab	6	8
Cholangioca	Amplification		PD	Trastuzumab	10	12
Gallbladder cancer	Amplification	PIK3CA, CDKN2A/B, TP53, ZNF703	MR	Lapatinib	15	19
Gallbladder cancer	Amplification	TP53	PR	Trastuzumab	>92	>92
Gallbladder cancer	Amplification	NRAS, PIK3CA, RB1,PTEN, TP53	PR	Trastuzumab	>40	>62
Gallbladder cancer	Amplification		PR	Trastuzumab	168	178
Gallbladder cancer	Amplification		PR	Trastuzumab	38	113
Gallbladder cancer	Amplification	FGFR3-TACC3 fusion, TP53, CCNE1, MCL1, MYC	SD	Trastuzumab + pertuzumab	+8	+8

*SD* stable disease, *PD* progressive disease, *MR* mixed response, *PR* partial response

## Discussion

*HER2/neu* gene is a key driver of tumorigenesis and its overexpression as a result of gene amplification is a critical target for therapy in breast cancer. Other solid tumors with reported overexpression of this gene include gastric adenocarcinoma (11 %), pulmonary adenocarcinoma (28 %), colorectal adenocarcinomas (17 %), pulmonary squamous (11 %), and pancreatic adenocarcinoma (7 %). There are several existing reports of HER2/neu overexpression in gallbladder cancer and the incidence has varied widely, depending upon the method used for assessment and scoring technique. Our group recently studied HER2/neu expression in 187 cases of gallbladder cancer; this is the largest reported series to date using the commonly accepted CAP/ASCO criteria. We noted than 13 % of patients have HER2/neu overexpression (3+ by immunohistochemistry). Yoshikawa and colleagues reviewed 236 cases of surgically resected cholangiocarcinoma and reported a 0.9 and 8.5 % incidence of HER2/neu expression in intra- and extrahepatic cholangiocarcinoma, respectively. In their series, early stage disease and well-differentiated tumors had a higher incidence of HER2/neu positivity. Data with regard to the prognostic value of HER2/neu overexpression in biliary cancers is mixed, with some studies suggesting a worse prognosis [23, 24], while others suggest the contrary [25, 26].

NGS for HER2/neu gene amplification may be regarded as the gold standard. However, this technology is dependent upon the quality of the pathological sample and DNA degradation may result from formalin fixation. In our retrospective series, the criteria for HER2/neu positivity were stringent and included 3+ protein expression on immunohistochemistry, FISH ratio higher than 2.2 or HER2/neu genetic aberration in a Clinical Laboratory Improvement Amendment (CLIA) compliant laboratory. Slamon and others have described the remarkable success of HER2/neu-targeted therapy for breast cancer [27–29]. In gastric cancer, the trastuzumab for gastric adenocarcinoma (ToGA) trial established the benefit of trastuzumab in combination with a fluoropyrimidine plus cisplatin in a randomized phase 3 trial of gastric cancer patients with overexpression or gene amplification of HER2/neu [30]. It is logical therefore to investigate HER2/neu targeting in other cancers that have gene amplification or overexpression.

Although the number of cases in this retrospective review is limited, to our knowledge, this is the largest reported case series of gallbladder cancer with *HER2/neu* amplification or overexpression treated with targeted therapy. Our experience demonstrates that HER2/neu-directed therapy appears to be beneficial for gallbladder cancer cases with *HER2/neu* amplification. Only one case in our series failed to respond, this tumor had *HER2/neu* mutation (V777L in the kinase domain). Two prior case reports have described responses with trastuzumab in subjects with gallbladder cancer. In the first, a 45-year-old woman received paclitaxel and trastuzumab and a dramatic regression of her lung metastases occurred [31]. In the second, a 61-year-old woman also with gallbladder cancer had a remarkable response to weekly paclitaxel and trastuzumab, which persisted when trastuzumab was continued as a single agent [32]. An estimated 80–100,000 cases of gallbladder cancer are diagnosed worldwide annually, and the benefit of targeted therapy for those with *HER2/neu* amplification would be substantial.

In this review, we also report a patient with gallbladder cancer with *FGF3-TACC3* fusion gene with negative *HER2/neu* gene amplification on NGS (confirmed by IHC and FISH, both being negative) who was treated with targeted FGFR inhibitors (dovitinib followed by pazopanib) resulting in initial response. She underwent repeat tumor biopsy on disease progression that then showed *HER2/neu* amplification on NGS (confirmed by IHC and FISH). In this case, we hypothesize that the *HER2/neu* amplification was a secondary event leading to resistance to FGFR inhibitors. Interestingly, FGFR2 addiction has been reported to be a mechanism leading to lapatinib resistance in breast cancer [33]. However, to our knowledge this is the first report of *HER2/neu* amplification as a mechanism of resistance to FGFR inhibitors. This finding supports the role of repeat tumor biopsy in the case of disease progression for re-assessment of HER2/neu status in select cases.

In contrast, trastuzumab was less effective in the case of cholangiocarcinoma and there were no responses noted in our series. Two of these cases with cholangiocarcinoma had *HER2/neu* mutations, which are not known to be responsive to trastuzumab, but may potentially respond to tyrosine kinase inhibitors like lapatinib, neratinib, and canertinib [34]. HER2/neu-targeted therapy has also not proven to be effective despite the presence of gene amplification in diseases like colon and pancreas cancers. The reason for this lack of efficacy is not known. Junttila et al. demonstrated that trastuzumab disrupts ligand-independent ErbB2/ErbB3/PI3K complexes blocking AKT signaling; when *PI3K* is mutated or dysfunctional, complex disruption does not inhibit *AKT* [35]. Another possible explanation could involve the higher rate of *KRAS* mutations seen in intrahepatic cholangiocarcinomas as compared with gallbladder cancers, possibly mediating resistance to upstream HER2 blockade [2, 5, 36].

In summary, HER2/neu-directed therapy is a promising avenue for patients with gallbladder cancer with gene amplification and should be further explored in an international clinical trial.
